# The Relation Between Temperature, Ozone, and Mortality in Nine French Cities During the Heat Wave of 2003

**DOI:** 10.1289/ehp.8328

**Published:** 2006-06-22

**Authors:** Laurent Filleul, Sylvie Cassadou, Sylvia Médina, Pascal Fabres, Agnés Lefranc, Daniel Eilstein, Alain Le Tertre, Laurence Pascal, Benoit Chardon, Myriam Blanchard, Christophe Declercq, Jean-François Jusot, Hélène Prouvost, Martine Ledrans

**Affiliations:** 1 Institut de Veille Sanitaire, Bordeaux, France; 2 Observatoire Régional de la Santé Ile-de-France, Paris, France; 3 Observatoire Régional de la Santé Nord Pas-de-Calais, Lille, France

**Keywords:** heat wave, mortality, ozone, temperature

## Abstract

**Background:**

During August 2003, record high temperatures were observed across Europe, and France was the country most affected. During this period, elevated ozone concentrations were measured all over the country. Questions were raised concerning the contribution of O_3_ to the health impact of the summer 2003 heat wave.

**Methods:**

We used a time-series design to analyze short-term effects of temperature and O_3_ pollution on mortality. Counts of deaths were regressed on temperatures and O_3_ levels, controlling for possible confounders: long-term trends, season, influenza outbreaks, day of the week, and bank holiday effects. For comparison with previous results of the nine cities, we calculated pooled excess risk using a random effect approach and an empirical Bayes approach.

**Findings:**

For the nine cities, the excess risk of death is significant (1.01%; 95% confidence interval, 0.58–1.44) for an increase of 10 μg/m^3^ in O_3_ level. For the 3–17 August 2003 period, the excess risk of deaths linked to O_3_ and temperatures together ranged from 10.6% in Le Havre to 174.7% in Paris. When we compared the relative contributions of O_3_ and temperature to this joint excess risk, the contribution of O_3_ varied according to the city, ranging from 2.5% in Bordeaux to 85.3% in Toulouse.

**Interpretation:**

We observed heterogeneity among the nine cities not only for the joint effect of O_3_ and temperatures, but also for the relative contribution of each factor. These results confirmed that in urban areas O_3_ levels have a non-negligible impact in terms of public health.

During August 2003, record high temperatures were observed across Europe, and France was the country most affected (Institut de Veille Sanitaire 2003a). Between 1 and 18 August, > 60% of the meteorological stations recorded temperatures > 35°C, and in 15% of the stations the temperature reached ≥ 40°C. In a recent report, Hémon and Jougla (2003) estimated that about 15,000 excess deaths occurred between 1 and 20 August. During the same period, elevated ozone concentrations were measured all over the country (European Environment Agency 2003). O_3_ is a photochemical air pollutant, and several studies have shown that daily variations in its levels are associated with daily variations in number of deaths after adjusting for the independent effect of temperature (Basu and Samet 2002).

Questions have been raised concerning the contribution of air pollution to the health impact of the summer 2003 heat wave. Some European authors have estimated the number of deaths attributable to O_3_ exposure during summer 2003. In the Netherlands, Fischer et al. (2004) showed an excess of 400–600 air pollution–related deaths. Others also carried out a health impact assessment in the United Kingdom (e.g., Stedman 2004). However, the authors of these studies (Fischer et al. 2004; Stedman 2004) applied preexisting exposure–response functions defined within usual conditions of temperature and O_3_ levels and did not take into account the exceptional meteorological conditions of summer 2003.

The aim of this study was, first, to estimate a new exposure–response function and the consequent health impact assessment between O_3_ and the risk of mortality in nine French cities, taking into account particularly the 2003 heat wave and compare the results with previous estimates (which excluded the heat wave) in the same cities (Le Tertre et al. 2002). The second objective was to estimate, for the 2003 heat wave period, the relative contribution of O_3_ in the joint excess risk of mortality related to temperature and O_3_ in each of the nine French cities.

## Materials and Methods

### Study area and environmental data

**Figure 1:**
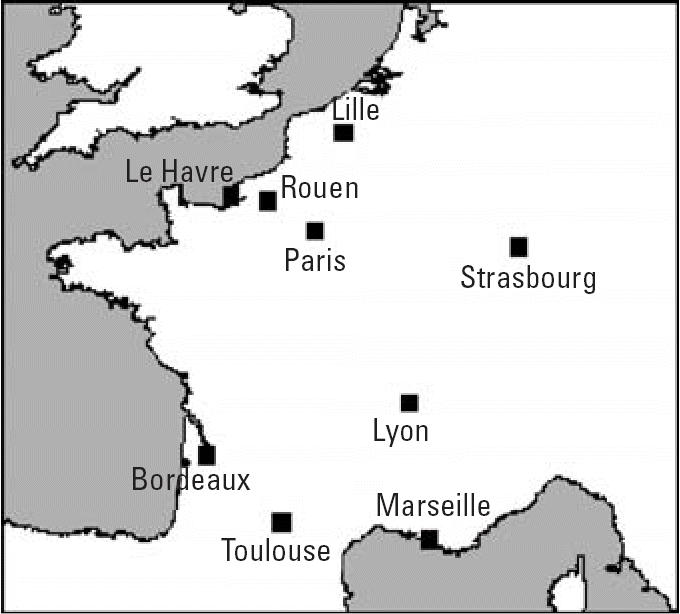
Location of the nine cities.

The nine cities involved in this study (Figure 1) are those participating in the French surveillance program for health risks related to urban air pollution (PSAS-9; Programme national de surveillance des effets sur la santé de la pollution de l’air dans 9 villes françaises).

In each city of the PSAS-9 program (Institut de Veille Sanitaire 2002), we defined a study area according to the localization of air pollution monitoring stations and daily population displacements. We obtained air pollution data from local air monitoring networks, which have measured O_3_ levels since 1996. France has a national standardized classification system for air quality monitoring stations. Urban or suburban monitoring stations used for the construction of the O_3_ indicator were selected according to the following criteria: The values measured by each station must be sufficiently correlated and close to the ones measured by every other station (correlation coefficient > 0.6, 75th percentile of the distribution of the values recorded by a station higher than the 25th percentile of the distribution of the values recorded by every other station). We constructed daily exposure indicators by calculating, for each day of the 1996–2003 period, the arithmetic mean of the 8-hr maximal concentrations recorded by each station selected. Then we calculated the mean of these values to obtain the daily indicator. Meteorological data (daily minimal and maximal temperature and relative humidity) were provided by Météo-France (http://www.meteo-france.fr/FR/index.jsp).

### Health data

The National Institute of Statistics and Economic Studies (INSEE 2005) provided mortality data. We considered daily counts of deaths in each study area that occurred during the 1996–2003 period. Influenza data were provided by the teleprocessing national network of monitoring and information on transmissible diseases (Valleron and Garnerin 1992), except for the Paris metropolitan area, where the influenza indicator was defined, during influenza epidemics (as defined by the Regional Group for the Observation of Influenza; GROG) (Cohen et al. 2005), as the daily number of doctor’s house calls for influenza symptoms, recorded by SOS Médecins, an emergency doctor house calls system (http://www.sosmedecins-france.fr/).

### Statistical analysis

We used a time-series design to analyze short-term effects of temperature and O_3_ pollution on mortality. Counts of deaths were regressed on temperatures and O_3_ levels, controlling for possible confounders: long-term trends, season, influenza outbreaks, day of the week, and bank holiday effects. We used Poisson regression models allowing for overdispersion and autocorrelated data. To characterize health effect of temperature and O_3_, the weather and air pollution variables included in models captured the expected effects, independent of the heat wave, of these variables on daily mortality. Temperature variables were modeled using penalized cubic regression spline. Temperature terms were modeled using 3 degrees of freedom for each (minimal temperature and maximal temperature).

We used thin plate regression splines to allow for potentially nonlinear effects and interactions between the variables. The degree of smoothing by the spline function was chosen to remove seasonal and long-term temporal trends and to minimize autocorrelation in the residuals. To compare the excess risks with our previous results, O_3_-8hr (O_3_ 8-hr mean) of the current and the previous days (0–1 day lag) was introduced as a linear term, following the APHEA-2 (Air Pollution and Health: A European Approach) methodology (Touloumi et al. 2004).

Because the impact of heat waves is usually related to small differences between night (minimum) and day (maximum) temperatures, we introduced both maximum and minimum temperatures in our models. We tested same-day, 1-day, 2-day, and 3-day temperature lags and their second-degree interactions to allow for a potential delay in temperature effects. The choice of temperature variables (minimum, maximum), lags (1 to 3 days), and interactions was based on Akaike’s information criterion (Akaike 1973). We also tested the potential interaction between O_3_-8hr and temperatures variables, and we introduced in the models a variable taking into account humidity levels (maximum daily relative humidity). However, this did not improve the quality of the models, as assessed by the value of the Akaike criterion.

For comparison with the previous results for the nine cities, we calculated pooled excess risk using a random effect approach (Institut de Veille Sanitaire 1999). Nevertheless, according to heterogeneity observed between the nine cities, we also estimated excess of risk using an empirical Bayes approach (shrunken estimates) (Post et al. 2001). In the presence of heterogeneity, city-specific estimates vary regarding the overall effect estimate for two reasons: *a*) the true heterogeneity in the estimates, and *b*) additional stochastic error. A city-specific estimate reflects the first source of variation but not the second one. The use of shrunken estimates allows reduction of the stochastic variability of the local estimates. All analysis was done using R (R Development Core Team 2004).

For each of the nine cities, we calculated the mean of daily excess risk (shrunken estimates) from 3 to 17 August 2003 for O_3_-8hr and temperature together, using as baseline O_3_-8hr and temperature levels for the same period of the three previous years. In this joint excess risk, the contribution of O_3_-8hr was calculated as the ratio between logarithm of O_3_-8hr relative risk and logarithm of joint O_3_-8hr and temperature relative risk. The O_3_-8hr excess risk during 3–17 August 2003 was also used to assess the health impact of O_3_ exposure during this period, according to the health impact assessment methodology described elsewhere (Institut de Veille Sanitaire 2003b).

## Results

The study population consisted of about 11 million people, as indicated by the 1999 census provided by INSEE (2005). Table 1 presents population sizes and mean daily counts of deaths for each city. O_3_ levels and temperature measured during summer 2003 are also described. The 50th percentile of the O_3_-8hr average ranged from 80 μg/m^3^ in Lille to 123 μg/m^3^ in Marseille (Table 1). The daily mean of maximal temperature ranged from 36.3°C in Le Havre to 40.7°C in Bordeaux. For minimal temperature, the daily mean ranged from 12.7°C in Le Havre to 19.8°C in Marseille (Table 1).

In each city, the excess risk of mortality associated with a 10-μg/m^3^ increase in the O_3_-8hr (0–1 day lag) is presented in Table 2 for the current study period (1996–2003) and the previous one (1990–1997) (Institut de Veille Sanitaire 2002). For 1996–2003, we observed a positive association between an increase in O_3_ levels and mortality in each city except Lyon. The relationship is significant except for Bordeaux, Lyon, and Le Havre. The pooled excess risk of death is significant [1.01%; 95% confidence interval (CI), 0.58–1.44] for an increase of 10 μg/m^3^ in O_3_ levels for the nine cities.

Compared with previous results obtained for the same cities and using the same analysis design, the pooled excess risk increased moderately between the two periods. However, local excess risk variations between the two periods differed according to the city: They increased in Le Havre, Lille, Rouen (becoming significant), and particularly Toulouse. They decreased in Marseille and Lyon and remained stable in Paris and Strasbourg. Globally, local excess risks were more heterogeneous among cities during the second period (1996–2003) than during the first period (1990–1997).

For the 3–17 August 2003 period, the excess risk of deaths linked to O_3_-8hr and temperatures together ranged from 10.6% in Le Havre to 174.7% in Paris (Table 3). When we compared the relative contributions of O_3_ and temperature to this joint excess risk, we observed that the contribution of O_3_ to mortality varied according to the city, ranging from 2.5% in Bordeaux to 85.3% in Toulouse. In Paris, Lyon, and Bordeaux, the temperature had a major effect during 3–17 August, and the relative contribution of O_3_ was low (< 8%). In Rouen, the temperature effect was also slightly more important (67%). Inversely, the percentage of O_3_ was greater in Strasbourg and Toulouse (> 75%). On the whole, we observed heterogeneity between the nine cities not only for the joint effect of O_3_-8hr and temperatures, but also for the relative contribution of each factor.

**Figure 2:**
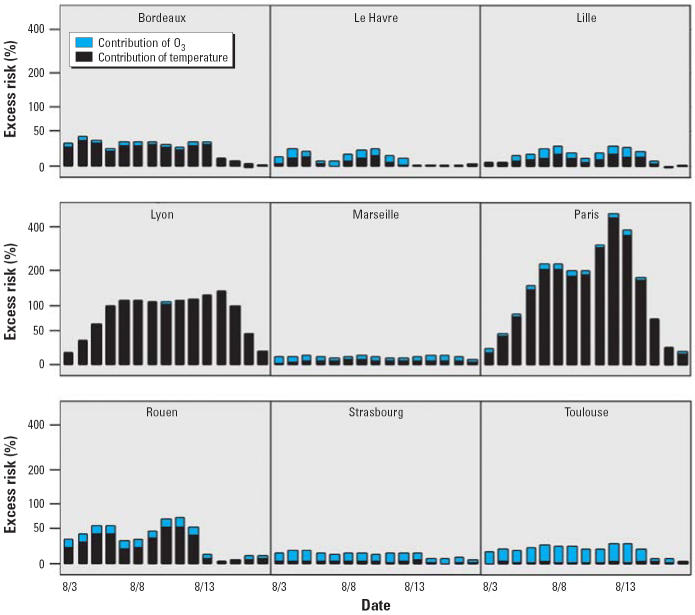
Daily values of excess risk of deaths linked to O_3_ and temperature and percentage of these two factors in all-ages population, for nine cities, August 2003, France.

In each city, the daily distribution of O_3_-8hr and temperature effects did not vary during the period. However, the daily variation of the excess risk linked to O_3_ and temperature together showed a temporal and spatial variability according to the city (Figure 2). In two cities (Rouen and Le Havre) variations showed two peaks of excess risk. In other cities (Paris, Lyon), we observed an increase between 3 and 7 August and then stability of excess risks until 15 August. For Bordeaux and Toulouse, excess risks were constant until 13 August and then decreased. For Strasbourg and Marseille, excess risks were stable during the entire period.

The rate of short-term death attributable to O_3_ exposure during 3–17 August 2003 ranged from 0.9 (Lyon) to 5.5 (Toulouse) for 100,000 inhabitants, leading to a total of 379 short-term attributable deaths during this period for the nine cities.

## Discussion

Our findings show a non-negligible impact of O_3_ during the heat wave: 379 short-term attributable deaths for the nine cities using as baseline O_3_-8hr levels for the same period of the 3 previous years. The present study does not allow us to identify the overall effects of temperature versus O_3_ because part of the temperature effect may be captured by the seasonal effect that was taken into account in the model. However, this study quantifies the very short-term effects of temperature and O_3_, and allows us to assess what happened during the heat wave. The correlations between O_3_ and temperature were quite high during the heat wave, but according to our statistical method, we have at least partly separated their respective effects. The observed correlations between O_3_ and minimum and maximum temperature, each adjusted on season and weekday effect, were respectively equal to 0.71 and 0.89 during the heat wave. These correlations are too high to separate the specific effect of each covariate. But on the previous years, which were included in the analysis, we also observed days with the same levels of O_3_ and not necessarily the same levels of temperature. The correlations when we select only the days > 108 μg/m^3^ (average level during the heat wave) of O_3_-8hr, are now respectively equal to 0.6 and 0.7. These correlations are still high but not so dramatic in terms of estimation.

Our analysis also showed a global significant relationship between O_3_ and excess risk of death in the combined nine cities for 1996–2003, including the heat wave. Compared with previous results for these cities (Institut de Veille Sanitaire 2002), the pooled estimate remained stable but local estimates varied according to the city.

Several hypotheses could explain these variations between the two studies. First, the study periods were different. The second included the 2003 heat wave period and also more recent data sets provided by more monitors than for the first period. Second, for the current analysis, we introduced two to four temperature variables and their interactions to capture their effects as much as possible; the previous models included only two temperature variables for controlling confounding effect of these factors. O_3_ was similarly taken into account in the two analyses: a linear term, mean of O_3_ levels for the current and previous days. The consequence of the new approach is a probable underestimation of O_3_ excess risks due to the *a priori* nature attributed to the relationship. Third, our analysis applied more strict convergence criteria than in the previous analysis, as suggested by Dominici et al. (2002). Also, to avoid the underestimation of parameters variance, we used R Programming Environment for Data Analysis and Graphics (R Development Core Team 2004). Finally, as suggested by recent discussions (e.g., Dominici et al. 2002) about optimal smoothing method, we used thin plate regression splines instead of LOESS smoothers used for previous analysis.

Numerous studies have shown associations between O_3_ levels and mortality. Short-term effects were the most studied, and typically an overall estimate (based on a meta-analysis) is provided. A recent meta-analysis (Anderson et al. 2004) is based on 15 European studies published between 1999 and 2002 about the effect of O_3_ on all-causes mortality. The pooled excess risk of death in all-ages population for an increase of 10 μg/m^3^ of O_3_ was 0.3% (95% CI, 0.1–0.4). The more recent results of the APHEA2 project (Gryparis et al. 2004) showed similar values: In summer, pooled estimates for the increase in the total daily number of deaths associated with O_3_-8hr increases of 10 μg/m^3^ (average of lags 0 and 1) was 0.34 (95% CI, 0.27–0.50) and 0.31 (95% CI, 0.17–0.52), respectively, for fixed effects and random effects. Our pooled excess risk is larger than these estimates: The issue of the impact of extreme levels of O_3_ and temperature on the results, even scarce, should be addressed.

As part of the APHEIS (Air Pollution and Health: A European Information System) program (APHEIS Group 2005), an analysis was conducted to address the issue of using alternative city-specific estimates: local estimate, shrunken estimate, estimate adjusted on effects modifiers, and overall estimate. This analysis was conducted on exposure to particles ≤ 10 μm in aerodynamic diameter, but its conclusion may be extended to other pollutant exposures. The use of the local estimate seems subject to too much noise to be really effective. On the other hand, applied to a single city, the overall estimate could not adequately reflect the heterogeneity present in the data. The two other derived city-specific estimates seem to give similar results. We preferred the shrunken estimate because it makes no inference about the relation with potential effect modifiers.

Regarding the health effect of heat waves, numerous studies also show adverse effects of high temperatures. Basu and Samet (2002) have reviewed epidemiologic evidence and reported that a number of studies have shown that mortality increases during heat waves. They observed that temperature at lag times of 0–3 days produce the maximum effect of mortality following heat waves.

During periods of high temperatures, our results show that health effects are different according to the city. These differences are coherent with previous results observed in France (Vandentorren et al. 2004) showing the same trend of mortality according to geographical area. Different factors may be responsible for these results. McGeehin and Mirabelli (2001) reported that the elderly and young children are particularly vulnerable to heat waves. Others risk factors—including poverty, social isolation, and certain medications connected with aging—were associated with an excess of death during heat waves (McGeehin and Mirabelli 2001; Semenza et al. 1996). Characteristics of the population of each city could therefore explain some of the differences observed in our results. Moreover, environmental characteristics of each city, such as the heat island effect, may also explain the heterogeneity of the results. Heat islands have been consistently shown to be associated with urban density. A strong relation exists between the population of a city and the temperature encountered in its dense center (Oke 1973; World Health Organization Europe 2004). The urban heat island effect influences the relation between the temperature measured in meteorological monitoring stations (data used in our models) and the temperature to which city inhabitants are really exposed. Hence, this could contribute to the difference in temperature effects observed across the nine participating cities.

In conclusion, these results confirmed that in urban areas O_3_ levels have a non-negligible impact for public health, even if this impact is low in terms of individual risks. Our findings also showed that the relative contribution of O_3_ and temperature in the high mortality during the 2003 heat wave was heterogeneous among cities according to local specific characteristics.

## Figures and Tables

**Table 1:** Population size, mortality, O_3_, and temperature in nine French cities during summer 2003 (1 June–30 September).

			O_3_ [8-hr mean) (μg/m^3^)]			Temp [daily mean (°C)]	
City	Population (no.)	Daily deaths (mean ± SD)	25th P	50th P	75th P	Minimum	Maximum
Bordeaux	584,164	13.6 ± 5.0	80	92	116	14.3	40.7
Le Havre	254,585	5.6 ± 2.7	69	82	100	15.3	36.3
Lille	1,091,156	21.8 ± 5.3	67	80	104	12.7	36.6
Lyon	782,828	19.4 ± 9.7	87	112	136	17.3	39.9
Marseille	856,165	23.8 ± 5.8	104	123	137	19.8	37.6
Paris	6,164,418	137.0 ± 107.0	70	93	122	16.3	39.3
Rouen	434,924	10.8 ± 4.4	72	88	111	12.8	37.9
Strasbourg	451,133	9.5 ± 3.6	86	119	148	14.5	38.4
Toulouse	690,162	13.3 ± 4.3	95	114	135	18.0	40.4

Abbreviations: P, percentile; Temp, temperature.

**Table 2:** Local, pooled, and shrunken excess risks (%) of mortality (all ages) for a 10 mg/m^3^ increase in O_3_ levels in the 9 cities, 1990–1997 and 1996–2003.

	1990–1997 study period	1996–2003 study period	
City	Local excess risk (95% CI)	Local excess risk (95% CI)	Shrunken excess risk (95% CI)
Bordeaux	—	0.58 (−0.37 to 1.54)	0.72 (−0.07 to 1.51)
Le Havre	0.61 (−0.56 to 1.79)	1.17 (−0.29 to 2.55)	1.09 (0.12 to 2.07)
Lille	0.27 (−0.59 to 1.14)	0.96 (0.30 to 1.61)	0.97 (0.37 to 1.56)
Lyon	0.16 (−0.55 to 0.87)	−0.02 (−0.71 to 0.67)	0.19 (−0.43 to 0.80)
Marseille	1.89 (0.90 to 2.89)	1.08 (0.46 to 1.72)	1.07 (0.50 to 1.65)
Paris	0.44 (0.17 to 0.71)	0.55 (0.28 to 0.83)	0.57 (0.30 to 0.84)
Rouen	0.82 (−0.14 to 1.79)	1.35 (0.28 to 2.42)	1.22 (0.38 to 2.07)
Strasbourg	1.08 (0.33 to 1.83)	1.12 (0.36 to 1.88)	1.09 (0.43 to 1.76)
Toulouse	0.74 (−0.22 to 1.70)	3.12 (2.09 to 4.17)	2.38 (1.55 to 3.21)
Pooled excess risk	0.66 (0.34 to 0.97)	1.01 (0.58 to 1.44)	NA

Abbreviations: —, not available for the first period; NA, not applicable.

**Table 3:** Excess risk (%) between 3 August and 17 August 2003 linked to O_3_ and temperature, percent of O_3_, and percent of temperature in the nine cities, France: mortality, all ages.

City	Excess risk O_3_ and temperature (%)	Percent of O_3_	Percent of temperature
Bordeaux	25.00	2.46	97.54
Le Havre	10.58	58.00	42.00
Lille	13.97	44.61	55.39
Lyon	87.74	2.57	97.43
Marseille	11.19	50.30	49.70
Paris	174.68	7.33	92.67
Rouen	35.24	32.60	67.40
Strasbourg	11.75	75.95	24.05
Toulouse	17.98	85.34	14.66
